# Evaluation of antimicrobial activity of chondrillasterol isolated from *Vernonia adoensis* (Asteraceae)

**DOI:** 10.1186/s12906-019-2657-7

**Published:** 2019-09-06

**Authors:** Winnie Mozirandi, Dexter Tagwireyi, Stanley Mukanganyama

**Affiliations:** 1 Department of Biochemistry, University of Zimbabwe, P.O. Box MP 167, Mt. Pleasant, Harare Zimbabwe; 2 School of Pharmacy, College of Health Sciences, University of Zimbabwe, P.O. Box A178, Avondale, Harare Zimbabwe

**Keywords:** *Vernonia adoensis*, Antibacterial, Biofilms, Chondrillasterol

## Abstract

**Background:**

Bacteria have developed resistance to most of the current antibiotics. There is evidence suggesting that plant-derived compounds have a potential for interacting with biological processes. One of the plants commonly used in African ethnomedicine is *Vernonia adoensis* from the Asteraceae family. The leaves of the plant have been reported to have antimicrobial activity. Hence, the aim of this study was to isolate the bioactive compounds from the leaf extract and evaluate their antibacterial activity on *Staphylococcus aureus, Klebsiella pneumoniae* and *Pseudomonas aeruginosa.* In addition, the effect of the isolated compound on biofilms of *P. aeruginosa* was determined.

**Methods:**

Isolation of phytochemicals from the leaves of *V. adoensis* was done using column chromatography. Preparative TLC was used to further isolate mixed compounds in the fractions. Nuclear magnetic resonance spectroscopy and mass spectrometry was used to identify the isolated pure compounds. The broth microdilution assay was carried out to evaluate the antibacterial activity of the isolated compound on *P. aeruginosa*, *S. aureus* and *K. pneumoniae.* Crystal violet staining technique was used to evaluate the effect of the isolated compound on biofilms of *P. aeruginosa.*

**Results:**

The compound isolated from *V. adoensis* was identified as chondrillasterol. Chondrillasterol exhibited 25, 38 and 65% inhibition of growth on *S. aureus*, *K. pneumoniae* and *P. aeruginosa* respectively. At 1.6 μg/mL chondrillasterol completely disrupted mature biofilm of *P. aeruginosa* while at 100 μg/mL the compound completely inhibited formation of biofilms of the bacteria.

**Conclusion:**

Chondrillasterol isolated from *V. adoensis* has antibacterial properties against *S. aureus*, *K. pneumoniae* and *P. aeruginosa.* The compound also has biofilm inhibition and disruption activity against *P. aeruginosa* biofilms. Thus, the active phytochemical could be a useful template for the development of new antimicrobial agents with both antibacterial and antibiofilm activity.

**Electronic supplementary material:**

The online version of this article (10.1186/s12906-019-2657-7) contains supplementary material, which is available to authorized users.

## Introduction

Bacteria have developed resistance to most of the currently used antibiotics [1], thus, making bacterial infections a major cause of mortality in the health care system [2]. There is evidence to suggest that bacterial infections are becoming difficult to treat because the pathogens are capable of developing biofilm which aids in host establishment, population expansion and in disease proliferation [3]. More than 60% of all the human bacterial infections have been attributed to the persistence of biofilm formation by the respective bacteria [4]. When bacteria form biofilms, the biofilm structure facilitates the survival of disease-causing pathogens even in hostile environmental conditions. It has been shown that the nature of biofilm structure and physiological attributes of biofilm forming organisms confer an inherent resistance to hostile conditions including antimicrobial agents such as antibiotics [5]. Hence, there is need to develop new antibacterial agents which can inhibit formation or destroy the mature biofilms and thus, increasing susceptibility of microbes to antibiotics.

There is an increasing interest in the use of medicinal plant-derived compounds as alternative antibacterial agents [6]. Plants have formed the basis of traditional medicinal systems that have been in existence for thousands of years, and continue to provide humanity with new remedies [7]. Some of the drugs which are widely used in clinical practice have been obtained from plants [8]. *Vernonia adoensis* is a plant which is commonly used in African ethnomedicine [9]. It is a herbaceous plant from the *Vernonia* genus belonging to the family Asteraceae which is the largest genus with close to 1000 species [10]. In East Africa the decoction of the roots is mixed with other trees for the treatment of heart and kidney problems [11]. In the Rift valley and Western part of Kenya, *V. adoensis* is used traditionally to treat symptoms of sexually transmitted diseases such as gonorrhea [11]. The leaves of the plant are also used in the treatment of the symptoms of malaria [12]. In Tanzania this plant has been traditionally used in African ethnomedicine for the treatment of fever and upper respiratory tract infections [9]. *V. adoensis* is native and commonly distributed throughout Zimbabwe where it is known in vernacular *Shona* language as *Musikavakadzi* [13].

Several studies have been carried out to evaluate the antimicrobial potential of *V. adoensis* with the ultimate goal of justifying the traditional use of the plant species or discovering drugs [14, 15]. The plant extracts have shown antibacterial activity against *Staphylococcus aureus, Pseudomonas aeruginosa*, *Bacillus cereus* and *Bacillus subtilis* in-vitro [16]*.* The plant leaves have been reported to have very high anti-plasmodial activity against *Plasmodium falciparum* [17]*.* Chimponda and Mukanganyama [18], reported that the leaves of *V. adoensis* had inhibitory activity against *Mycobacterium aurum* and *Corynebacterium glutamicum.* There are studies which have been done to isolate phytochemicals from some *Vernonia* species and the compounds isolated were shown to have biological activities [19–21]. *V. adoensis* has been shown to possess some important pharmacological phytochemicals [21] but there is limited information on the biological activity of the specific compounds isolated from the plant. Most of the biological activity tests from the various studies were conducted using crude extracts obtained from different parts of the plant*.* Hence, the need to isolate phytochemicals from *V. adoensis* as they may yield lead compounds that have significant antimicrobial potential against selected strains of bacteria*.* Studies have shown that bacteria such as *P. aeruginosa, S. aureus and K. pneumoniae* are among the major causes of nosocomial infections that have produced a poor prognosis in the hospital [22]*.* From the advent of antimicrobial application in treatment of bacterial diseases, pathogenic bacteria have responded by developing varied forms of resistance [23]. *Klebsiella pneumoniae* has become resistant to carbapenem antibiotics which are often the last line of defense against Gram-negative infections [24]. *Staphylococcus aureus* strains are now resistant to penicillin [25]. In most Asian countries, 70–80% of the same strain have become methicillin-resistant [25]. *Pseudomonas aeruginosa* infections are now responsible for most nosocomial infections in hospitals and healthcare centers because there are no effective antimicrobial agents against it [26]. Also infection by *P. aeruginosa* through biofilm in lungs of cystic fibrosis (CF) patients is causing high morbidity and functional failure of this organ [27].

There is need for new antibacterial agents as bacteria have developed resistance to most of the currently used antibiotics [1]. In addition, current antibiotics have considerable limitations in terms of antimicrobial spectrum and side effects [28]. The indiscriminate use and misuse of antibiotics has led to increasing clinical resistance of previously sensitive microorganisms and to the occurrence of uncommon infections. Plant-based natural products have been found to be a rich source of antimicrobial agents [29]. Traditional medicinal plants have received significant attention as a source of new chemical entities because their phytochemicals may lead to new leads in drug discovery [30]. The aim of this study was to isolate the phytochemicals found in *V. adoensis* and evaluate their antimicrobial activity since previously the crude extracts were shown to have high antibacterial activities [31].

## Materials and methods

### Permission to use the plant

The collection and use of the plant for the study was approved by the ethical committee of the Faculty of Science Higher Degrees, University of Zimbabwe, paper HD/166 of 2016.

### Plant material collection

*Vernonia adoensis* was collected in Centenary, (Geographic coordinates, Latitude: 16°43′22″ S, Longitude: 31°06′52″ E, elevation above sea level: 1156) Mashonaland Central Province of Zimbabwe. Plant collection was done during the month of March 2018. Authentication and classification of the plant was done by a taxonomist from the National Herbarium and Botanic Garden (Harare, Zimbabwe). Herbarium samples C1 E7, were kept at the National Herbarium and Botanic Garden (Harare, Zimbabwe) and the Department of Biochemistry, University of Zimbabwe.

### Preparation of acetone extract

A previous study of evaluation of antibacterial activity of extracts from *V. adoensis* had shown the acetone extract to possess the most potent antibacterial activity [31] hence, the extract used in this study was prepared using acetone. *V. adoensis* leaves were washed under running tap water and dried in the oven at 40 °C. The dried leaves were pounded in a clean mortar and further ground in a two speed blender (Cole Parmer Instrument CO.,Vernon Hills, USA) to obtain a fine powder (4 kg) to which acetone (Sigma-Aldrich, Taufkirchen, Germany) was added to extract the photochemical by maceration [32]. The mixture was left for 72 h shaking on a magnetic stirrer for effective extraction of the plant components. The extract obtained was then filtered using WHATMAN’s no 1 filter paper (Sigma-Aldrich, Taufkirchen, Germany) into sterile beakers and the filtrate concentrated to dryness by evaporation at room temperature in a fume hood with an air stream. The dry extract was stored at 4 °C for further use.

### Bacteria and culture conditions

*Staphylococcus aureus* (ATCC 9144) and *Pseudomonas aeruginosa* (ATCC 27853) were obtained from the Division of Microbiology, Department of Biological Sciences at the University of Botswana. A clinical strain of *Klebsiella pneumoniae* were obtained from Parirenyatwa Hospital (Department of Medical Microbiology College of Health Sciences, Harare, Zimbabwe). Bacteria were inoculated into tryptic soy broth media and incubated overnight at 37 °C with shaking at 2 g-force in a Lab-Companion incubator (SI300 Incubated shaker, Jeio Tech, Korea). The bacterial cultures were centrifuged at 2068 g-force for 4 min in a Hettich Rotofix 32 centrifuge (Tuttlingen, Germany) and the supernatant discarded. The pellets were washed twice in phosphate-buffered saline and suspended in fresh media. The cells were standardised according to 0.5 McFarland standards to create inoculum densities of 2 × 10^6^ cfu/mL for use in the antibacterial activity determination assay, and bacterial broth cultures of 5 × 10^8^ cfu/mL for biofilm assays.

### Isolation of compounds

Isolation of compounds from the crude extract was done by column chromatography. The dried acetone extract (156 g) was dissolved in minimum quantity of distilled methanol and warmed in a water bath at 97 °C until completely dissolved. The solubilised extract was adsorbed on silica gel (60) and dried at room temperature. The column (6 × 90 cm) was prepared using silica gel (60–120 mesh) suspended in n-hexane. The extract adsorbed to silica was ground to powder and then poured onto the stationary phase. The column was run by gradient elution technique using hexane, ethyl acetate and methanol. A total of 450 fractions each with 250 mL were collected and characterised by TLC on silica gel plates. The spots developed were visualised at 254 and 365 nm using A425/G Allen Ultraviolet light (P. W. ALLEN & Co., London, UK). The TLC plate was sprayed with 5% sulphuric acid and incubated for 10 min at 120 °C. The fractions which showed the same TLC development profiles were pooled and allowed to dry at room temperature till the solvent evaporated. The fractions were placed on filter paper over a conical flask and washed to remove impurities. Washing of fractions was done using a solvent of higher polarity relative to the eluent. The washed compounds were left to dry at room temperature and thin layer chromatography was carried out to determine the purity of the dried compounds. All the fractions except for fraction 10 were in minute quantities such that no further analysis could be done with them. Fraction 10 was further purified using preparative TLC using 20 × 20 cm TLC silica gel 60 glass plates (Sigma-Aldrich, St. Louis Missouri, USA). Two bands were visible under UV at 254 nm and 366 nm and these were scrapped into separate beakers and labelled F10b1 and F10b2. To extract the compounds from the silica gel, they were placed on a filter paper (Whatman # 1, Sigma-Aldrich, Darmstad, Germany) on a funnel and chloroform was used to dissolve them. The purity of the compounds in the filtrate was checked by TLC analysis. The compounds eluted and collected as filtrates F10b1 and F10b2 were air dried and stored in vials (Chromacol clear 2 mL S/T vials, Sigma-Aldrich, St. Louis Missouri, USA) for further use.

### Spectral identification of compounds

NMR spectroscopic analysis were carried out at the Institute of Chemistry Potsdam, Germany. Samples F10b1 and F10b2 were dissolved in CD_2_Cl_2_ then 1D and 2D NMR spectra were recorded on a BRUKER AVANCE 500 spectrometer (Billerica, Massachusetts, USA). Residual solvent peaks of CD_2_Cl_2_ were used as a reference. Agilent HPLC 1260 System coupled to an Agilent Q-TOF 6530 Mass spectrometer was used to confirm the formula of the compound.

### Antibacterial activity of chondrillasterol

Chondrillasterol (Fig. 3) was identified as one of the compounds that were isolated from *V. adoensis*. The antibacterial activities of this compound against *P. aeruginosa, S. aureus* and *K. pneumoniae* were determined in-vitro using the broth microdilution method following guidelines from [33]. Two-fold serial dilutions of the pure compound from (12.5 to 100 μg/mL) were prepared and a volume of 100 μL was separately added to the wells of a 96-well microplate. To each of the wells 100 μL of bacterial cells were added to give a final concentration of 1 × 10^6^ cfu/mL. Ten two-fold serial dilutions of ciprofloxacin (0–100 μg/mL) as well as levofloxacin were also prepared and 100 μL added to wells of the plate containing an equal volume of cells. Ciprofloxacin was added to *P. aeruginosa* and *S. aureus* cells while levofloxacin was added to wells with *K. pneumoniae*. The wells with tryptic soy broth served as the sterility control and wells with cells only served as the negative control. Pre-incubation absorbance readings of the plate were measured at 590 nm using the microplate reader (Tecan Genios-Pro microplate reader, Grödig, Austria) and the plate was incubated overnight without shaking at 37 °C in a LAB Doctor Mini Incubator (MID SCI, USA). The cell density was also determined after 20 h of incubation. Viability of cells after exposure to the extract was further evaluated by addition of 20 μL aliquots of 2 mg/mL 3-(4,5-dimethylthiazol-2-yl)-2,5- diphenyltetrazolium bromide (MTT) to each of the wells of the plate and incubating the plate at 37 °C for 2 h. Viable bacteria reduce the yellow MTT dye to a purple formazan [34].

### Effect of chondrillasterol on nucleic acid leakage

Evaluation of the ability of chondrillasterol to disrupt *P. aeruginosa* cells and cause leakage of nucleic acids was done according to the method of El-Nakeeb [35] with some modifications. *P. aeruginosa* (ATCC 27853) was grown overnight. The overnight culture was centrifuged at 2068 g for 4 min in a Hettich Rotofix 32 centrifuge (Tuttlingen, Germany) and the supernatant discarded. The pellet was suspended in 0.9% sterile saline until (OD_600_ = 1.5). The suspensions were exposed to 100 μg/mL and 50 μg/mL of chondrillasterol and incubated at 37 °C with shaking (2 g-force) for 10 min in a Lab-Companion incubator (SI300 Incubated shaker, Jeiotech, Korea). The controls used were cells exposed to 0.1% SDS for the positive control and untreated cells served as the negative control. From each sample, 1 mL aliquots were centrifuged at 20427 g-force for 1 min (Hettich Rotofix 32 centrifuge, Tuttlingen, Germany). The pellet was washed with 0.9% saline solution and suspended in 3 mL of saline. A volume of 3 μL propidium iodide were added to each sample and the solution was mixed. The samples were kept in the dark for 10 min after which fluorescence was measured at excitation and emission wavelengths of 544 nm and 612 nm respectively using an *f*max microplate spectrofluorometer (Molecular Devices, Sunnyvale, USA).

### Effect of chondrillasterol on formation of biofilm by *P. aeruginosa*

**Fig. 1:**
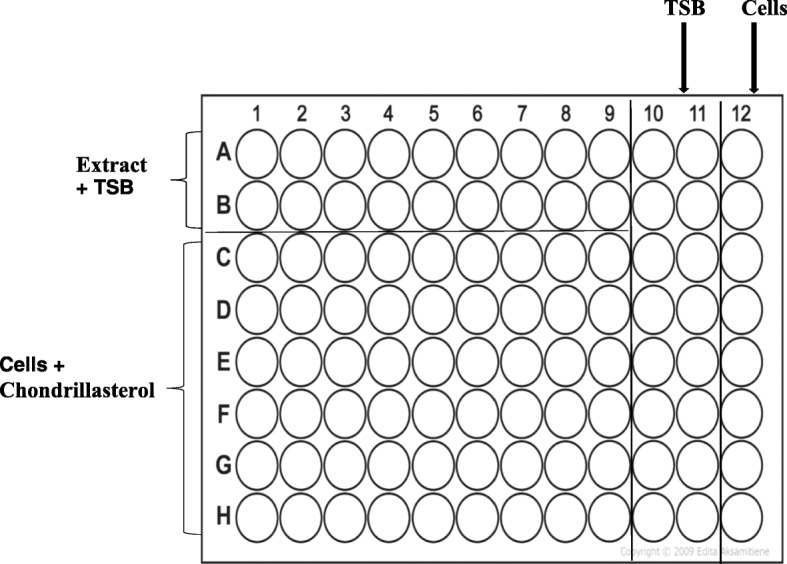
Plate layout for testing effect of chondrillasterol on mature biofilm. A two-fold serial dilution was carried out for chondrillasterol starting from 200 μg/mL. Increasing concentrations of chondrillasterol were added starting from column C1. Controls included: (i) the extract being incubated alone (rows 1 and 2), (ii) media alone (column 10 and 11) and (iii) viable bacterial cells incubated alone (column 12)

Evaluation of the ability of the isolated compound which had been identified as chondrillasterol to inhibit formation of *P. aeruginosa* biofilms was carried out using microtiter plate method according to Stepanovic [36] with slight modifications. An overnight culture of *P. aeruginosa* cells in tryptic soy broth supplemented with 1% glucose was centrifuged at 2068 g-force for 4 min. The inoculum was standardised according to 0.5 McFarland standards to a working suspension corresponding to 5 × 10^8^ cfu/mL. Double serial dilutions of chondrillasterol were performed to cover the range from 0.4 to 100 μg/mL as a final concentration in the wells. To detect the effect of chondrillasterol on biofilm formation, 100 μL of appropriate concentrations of the compound were added to six individual wells of a sterile 96-well polystyrene microplate (Greiner 96 well plates, Sigma-Aldrich, Germany) prior to inoculation. An equal volume of the numerically standardised *P. aeruginosa* bacterial suspension were added. The assay was carried out according to the plate lay out shown in Fig. 1. As a negative control to determine background optical density (OD) the first two rows of the plate contained 100 μL uninoculated medium added to an equal volume of the compound. In column 11 of the plate, 200 μL of bacterial cells only were dispensed and served as the positive control.

Aliquots of media (TSB + 1% glucose) were dispensed into each of eight wells of column 12 of microtiter plate to serve as a negative control. The plate was incubated in a non-shaking incubator at 37 °C for 72 h (Labcon orbital incubator, Labotec Co., Cape Town, South Africa). After incubation medium was decanted into a discard container and wells of the plate were washed twice with 0.9% sterile saline to remove planktonic bacteria. The contents of wells were emptied by inversion and plate dried upside-down for 15 min on absorbent paper in a biosafety cabinet (BioFlow-11, Labotec Co., Cape Town, South Africa). The plate was placed in an oven at 60 °C for an hour to fix the formed biofilm after which 0.1% crystal violet stain was added to each well and the plate incubated at room temperature for 20 min. Excess stain was rinsed off by decantation and the plate was washed three times with distilled water and left to air dry. After the plate had completely dried, the wells of the plate were filled by 200 μL of 95% ethanol to release the dye from the cells. The density of stained adherent bacteria was determined at 590 nm using EL × 800 Tecan Genios-Pro microplate reader (Grödig, Austria). The percentage inhibition of biofilm formation was calculated using the formula in Eq. 1:

1 $$ \%\mathbf{Biofilm}\ \mathbf{inhibition}=\left[\left(\mathbf{AB}-\mathbf{EF}\right)/\mathbf{G}\right]\times \mathbf{100}. $$

where AB is the optical density of the stained attached bacteria, EF is the optical density of the stained control cultures without microorganisms, and G is the optical density of the bacteria in suspended culture.

### Effect of chondrillasterol on mature biofilm of *P. aeruginosa*

The effect of chondrillasterol on mature biofilms was determined on 96-well microplates [36]. An overnight culture of *P. aeruginosa* cells was numerically standardised as above and 100 μL volume added to each of 6 wells of a polystyrene microplate (Greiner 96 well plates, Sigma-Aldrich, Germany). As a negative control to determine background optical density (OD) the first two rows of the plate contained 100 μL uninoculated medium added to an equal volume of the compound. In column 11 of the plate, 200 μL of bacterial cells only were dispensed and served as the positive control. Aliquots of media (TSB + 1% glucose) were dispensed into each of eight wells of column 12 of microtiter plate to serve as a negative control. The plate was incubated at 37 °C for 72 h without shaking in LAB Doctor Mini Incubater (MID SCI, USA) to allow for development of a mature biofilm. After incubation medium was aspirated with a pipette and the plate washed twice with 0.9% sterile phosphate buffered saline to remove non adherent cells. Serial dilutions of chondrillasterol ranging from 100 to 0.8 μg/mL were prepared and added to the wells with the preformed biofilm. The plate was incubated for 24 h at 37 °C without shaking. After incubation, the contents were decanted into a discard container and the plate was washed three times with sterile phosphate buffered saline to remove free-floating non-adherent cells. The plate was dried upside-down for 15 min on absorbent paper in the biosafety cabinet and placed in an oven (Mermmert, Schwabach, Germany) at 60 °C for an hour to fix the formed biofilm. The amount of biofilm that remained on the plate after exposure of a preformed biofilm to different concentration of the compound was quantified by crystal violet (CV) staining as described in earlier. The controls used were similar to those used in the assay to determine effect of the compound on formation of biofilm by *P. aeruginosa*.

### Statistical analysis

The data of the results obtained in this study were analysed using GraphPad Prism 5 for Windows (GraphPad Software Inc., San Diego, California, USA) version 5.03. The one-way analysis of variance test (ANOVA) with Dunnett’s Multiple Comparison Test was used to determine the level of significance where all treated samples were compared to the control. The values with *P* < 0.05 were considered statistically significant.

## Results

### Isolation of compounds

**Fig. 2:**
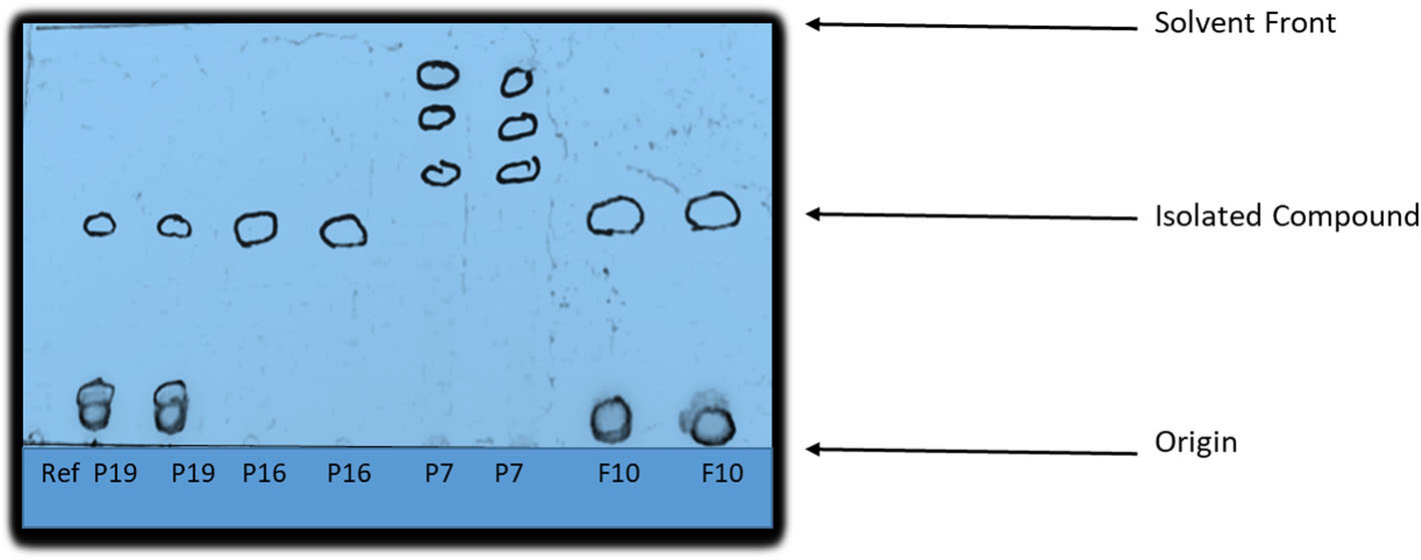
TLC profile of compounds from column chromatography. Single spot represents a pure compound while more than one spot represents a mixture of compounds isolated from *V. adoensis*

**Fig. 3:**
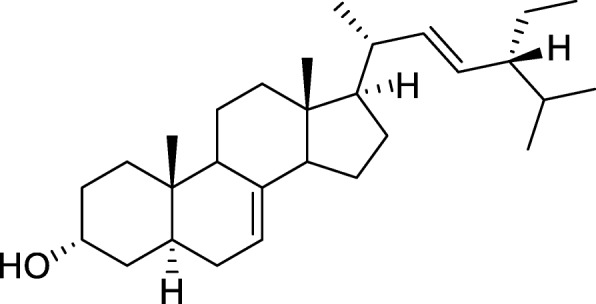
Structure of Chondrillasterol. Data to elucidate the structure was obtained by NMR and MS studies

Column chromatography of the acetone extract resulted in 450 fractions being collected. These fractions were combined into 20 pools in which most pools had a mixture of many compounds which were not pure. Only two pools labelled P19 and P7 had TLC profile showing the presence of three compounds while F10 and P16 had two and one compound respectively as shown in Fig. 2. The presence of a single spot from P16 implied that a pure compound had been isolated. However, no further analysis was done as the compound was in minute quantity. Further separation of compounds in the pool labelled F10 led to the isolation of two pure compounds which were labelled F10b1 and F10b2. Spectrometric analysis of the pure compound in F10b2 revealed that the compound was a wax. Outlined below is the data for the compound in F10b1: ^1^H NMR (CD_2_Cl_2_, 500 MHz), (ppm): 5.23 (1H, dd, J = 8.5 Hz, H-22), 5.21 (1H, m, H-7), 5.10 (1H, dd, J = 8.5 Hz, H-23), 3.58 (1H, m, H-3), 1.07 (3H, d, J = 6.5 Hz, H-21), 0.89 (3H, d, J = 6.5 Hz, H-27), 0.85 (3H, t, J = 6.0 Hz, H-29), 0.84 (3H, s, H-26), 0.83 (3H, d, J = 6.0 Hz, H-19), 0.59 (3H, s, H-18) ^13^C NMR (CD_2_Cl_2_, 125 MHz), (ppm):139.54 (C-8), 138.29 (C-22), 129.37 (C-23), 117.39 (C-7), 70.80 (C-3), 55.90 (C-14), 55.09 (C-17), 51.28 (C-24), 49.45 (C-9), 43.22 (C-13), 40.85 (C-20), 40.28 (C-5), 39.46 (C-12), 37.11 (C-1), 34.16 (C-16), 31.92 (C-25), 31.51 (C-2), 29.68 (C-6), 29.64 (C-16), 25.39 (C-28), 22.99 (C-11), 21.25 (C-15), 21.13 (C-21), 20.84 (C-26), 18.73 (C-27), 12.78 (C-19), 12.02 (C-29), 11.78 (C-18). Spectrometric analysis of the compound showed that F10b1 was chondrillasterol and the structure is shown in Fig. 3 with NMR data provided as suplementary material (see Additional file 1: Figure S1). The proton and carbon-13 NMR spectra were comparable to published literature ([37]. Further antimicrobial assays were done using chondrillasterol.

### Antibacterial activity of chondrillasterol

**Fig. 4:**
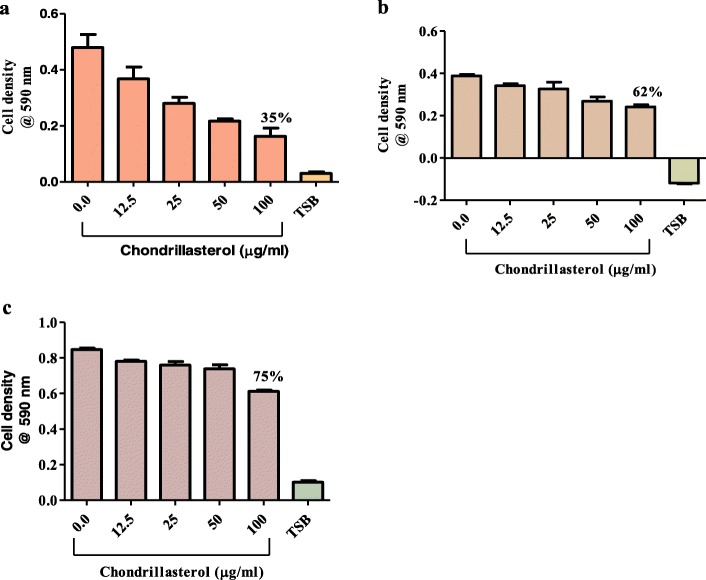
The effect of chondrillasterol on growth of bacteria pathogens. The pathogens were susceptible to chondrillasterol and (**a**) is % remaining viable cells *for P. aeruginosa*, and (**b**) is % of remaining viable cells for *K. pneumoniae* while (**c**) is % of remaining viable cells for *S. aureus* following exposure to chondrillasterol. Concentrations of 1.6 × 10^6^ cfu/mL of bacteria were used. Values are expressed as mean cell density at 590 nm wavelength ± the standard deviation (*n* = 4). TSB is tryptic soy broth

In-vitro tests were carried out to evaluate antibacterial activities of the isolated compound on *P. aeruginosa*, *S. aureus* and *K. pneumoniae*. Chondrillasterol was shown to possess antibacterial activity against all the tested strains (Fig. 4).

The most susceptible bacterium was *P. aeruginosa* whose growth was inhibited by 65% (Fig. 4a). Chondrillasterol also inhibited the growth of *K. pneumoniae* and *S. aureus* by 38% (Fig. 4b) and 25% (Fig. 4c) respectively.

### Effect of chondrillasterol on nucleic acid leakage

**Fig. 5:**
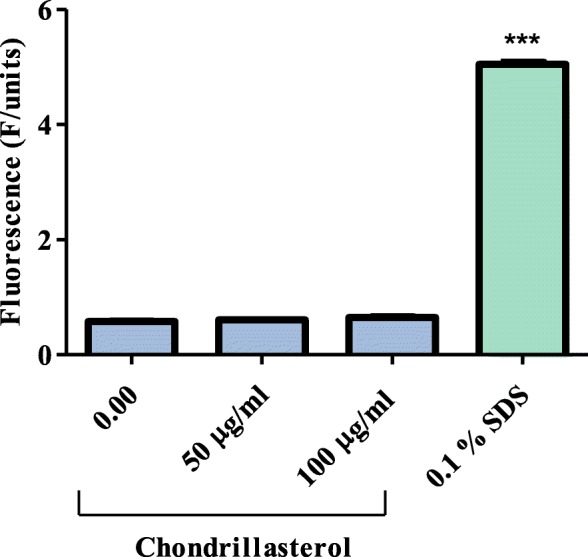
The effect of chondrillasterol on nucleic acid leakage in *P. aeruginosa.* Cells and control (0.1% SDS) were exposed to chondrillasterol in triplicate and the nucleic acid leakage was quantified. The test for significance was carried out by comparing the fluorescence of the samples to untreated cells (cells only), ***P < 0.0001. SDS: sodium dodecyl sulphate

Quantification of nucleic acids after exposure to chondrillasterol was performed to evaluate if the compound had the ability to interfere with the integrity of *P. aeruginosa* membrane and cause leakage of nucleic acid from the bacteria. It was shown that 100 and 50 μg/mL caused nucleic acid leakage of 0.650 and 0.606 F/units from *P. aeruginosa* respectively (Fig. 5). There was no statistically significant difference between nucleic acids leaked from cells exposed to chondrillasterol and nucleic acids leaked from untreated cells (0.578 F/units). Sodium dodecyl sulphate (SDS) caused significant leakage of nucleic acids from *P. aeruginosa* and the fluorescence of PI from cells exposed SDS was 5.047 F/units.

### Effect of Chondrillasterol on formation of biofilm

**Fig. 6:**
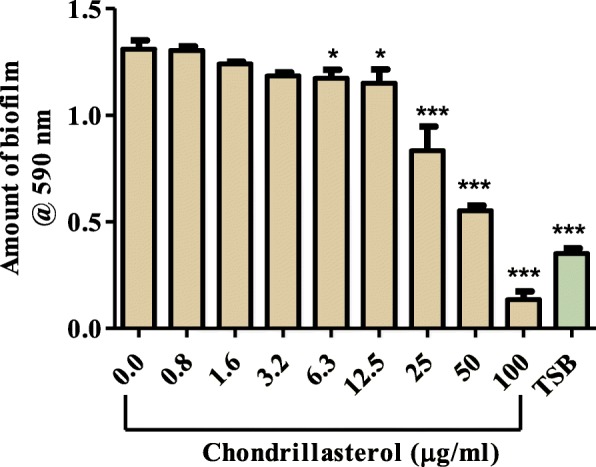
Effect of chondrillasterol on *P. aeruginosa* biofilm formation. *P. aeruginosa* (1.6 × 10^6^ cfu/mL) was incubated with different concentrations of the pure compound for 72 h. at 37 °C and the resulting biofilm mass was stained using crystal violet staining. The dye was extracted using ethanol and quantified spectrophotometrically at 590 nm. The absorbance values of crystal violet are directly proportional to the amount of biofilm that had formed. The error bars indicate the standard deviation from mean (*n* = 4). Dunnet multiple comparison test was used to compare columns using GraphPad Prism (GraphPad Software Inc., San Diego, California, USA) version 5.03. The asterisks (*) indicate statistically significant differences with the positive control (0.0) which represented cells only without extract * < 0.05 *** < 0.0001. TSB is tryptic soy broth.

*P. aeruginosa* cells were allowed to form biofilms in microplate in the presence and absence of chondrillasterol. Biofilm mass that was formed after 72 h of incubation was stained with crystal violet and the results are shown in Fig. 6.

There was a significant difference in the biofilm formed by bacteria exposed to chondrillasterol from 6.3 μg/mL and the biofilm formed in bacteria not exposed to the compound (cells only). The results also show that at 100 μg/mL chondrillasterol completely inhibited the formation of biofilms in microplates by *P. aeruginosa*.

### Effect of chondrillasterol on disruption of biofilm

**Fig. 7:**
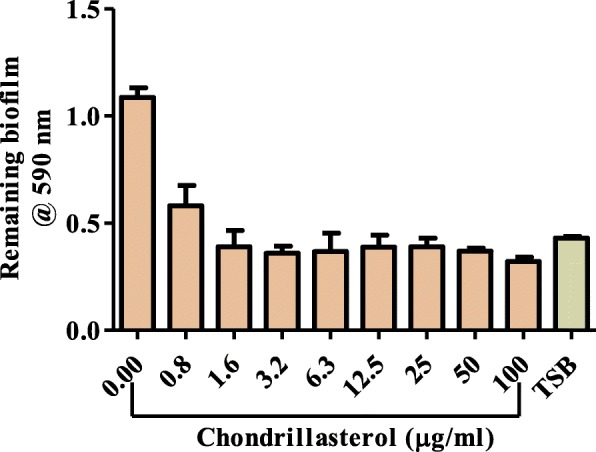
Effect of chondrillasterol on preformed biofilms by *P. aeruginosa*. Cells were incubated for 72 h. at 37 °C. Planktonic cells were aspirated and different concentrations of chondrillasterol added to the preformed biofilm. The plate was further incubated for 24 h. after which it was washed to remove planktonic cells. Remaining biofilm mass was stained using crystal violet staining and quantified at 590 nm. The absorbance values of crystal violet are directly proportional to the amount of biofilm that remained intact after exposure to extract. The error bars indicate the standard deviation from mean (*n* = 4). TSB is tryptic soy broth

Static biofilm quantification using the crystal violet method was performed to evaluate the effect of chondrillasterol on preformed *P. aeruginosa* biofilms in microplates. From the data shown in Fig. 7, the results show that all the tested concentrations of the isolated compound significantly disrupted preformed *P. aeruginosa* biofilms. The minimum concentration of chondrillasterol which disrupted *P. aeruginosa* biofilms was 1.6 μg/mL (Fig. 7).

## Discussion

A number of bioactive plant secondary metabolite compounds have been isolated from crude extracts and their structures were elucidated [38]. In this study, phytochemical investigation on the acetone extract of the leaf parts of *V. adoensis* resulted in isolation and structure elucidation of one compound; chondrillasterol. There are studies which have been done and also resulted in isolation of pure compounds from *V. adoensis* [39]. However, to the best of our knowledge this is the first time that the compound chondrillasterol has been isolated from this plant. Chondrillasterol had, however, been previously identified from *Agaratum fasigiatum* which is from the same family (Asteraceae) with *V. adoensis* [40]. Chondrillasterol had also been isolated from other species including *Gambeya boiviniana Pierre* [37] *and Lagenaria leucantha var.gourda* [41]. Isolation and identification of bioactive compounds found in crude plant extracts as building blocks for new antibiotics provides unique opportunities for the development of new effective antibiotics. In this study the bioactivity of the isolated compound was evaluated against the bacterial species; *S. aureus, P. aeruginosa* and *K. pneumoniae.* Antimicrobial susceptibility was observed with the isolated compound which had an inhibitory effect on the growth of all the tested strains. These results are in agreement with similar studies which have been done and shown that compounds isolated from plants have antibacterial activity against microbial pathogens [41]. Chondrillasterol exhibited lower antibacterial activity than that reported for the crude extract [31]. This might mean that the extract had compounds that exerted their antibacterial effect in a synergistic manner. Though the three microorganisms were sensitive to chondrillasterol, the most sensitive organisms were the Gram-negative bacteria, *P. aeruginosa* followed by *K. pneumoniae*. There are reports in literature that Gram-negative bacteria are less susceptible to antimicrobial agents because of the presence of the outer membrane which acts as a permeability barrier [42, 43]. The isolated compound is lipophilic which may result in it being able to penetrate the membrane barrier by dissolving in the lipid bilayer. Antibiotics which have been reported to be effective against the Gram-negative bacteria traverse the outer membrane by using the diffusion pathway through the lipid components of the outer membrane. They also traverse the membrane through porins or selective channels formed by specific beta-barrel proteins [44]. It is possible that chondrillasterol may be penetrating the membrane barrier using similar mechanism of action against *P. aeruginosa* and *K. pneumoniae.* However, the mechanism of action to effectively penetrate the membrane barrier may also be different from that being used by the current antibiotics. Previous studies have shown that natural products of higher plants may serve as sources of antimicrobial agents with possibly novel mechanisms of action [45, 46]. Antibacterial agents have also been shown to use different modes of action against bacterial pathogens, including altering membrane, protein leakage, and nucleic acid leakage [47]. To investigate the antibacterial mechanism of action of the isolated compound on the most susceptible bacteria, the ability of the isolated compound to disrupt *P. aeruginosa* membrane was determined. The results showed that chondrillasterol did not cause any leakage of nucleic acids from *P. aeruginosa* and may mean that the compound may be using a different mechanism of action and not killing the bacteria by causing leakage of nucleic acids. The antibacterial activity of the pure compound was, however, found to be lower than that of the standard antibiotic ciprofloxacin. The use of natural compounds with antibacterial activity is now increasingly being encouraged since natural products are perceived to be safe and mitigate many of the side effects that are often associated with conventional antimicrobials [6, 48].

Compounds isolated from plants have been shown to exhibit antibiofilm activity [49]. Clinically, biofilm inhibitors can be used directly to reduce virulence factors from infectious bacteria [50] or to treat infectious biofilm along with conventional antibiotics [51]. In this study, the antibiofilm potential of the isolated compound was evaluated against *P. aeruginosa*. *P. aeruginosa* is a ubiquitous bacterium in nature and this opportunistic pathogen can colonise various surfaces by forming a biofilm in which bacterial cells stick together and are embedded within a self-produced extracellular polysaccharide matrix [52, 53]. Cells which are in a biofilm are reported to be more resistant to antibiotics and biocides than planktonic cells, which often cause difficulties in eradicating them from individuals infected with the bacterium [54]. Chondrillasterol inhibited formation of biofilm by *P. aeruginosa*. The inhibition of biofilm formation by chondrillasterol was found to be concentration-dependent. An increase in concentration of chondrillasterol caused increased suppression of bacterial growth and this suggests that suppression of biofilm formation by the compound may be resulting from the inhibition of bacterial growth. The ability of a natural product to completely inhibit biofilm formation suggests that it would be a good candidate for use in controlling biofilm growth. Such compounds with anti-biofilm activities may be used directly to reduce virulence factors from infectious bacteria [50] or to treat biofilm along with conventional antibiotics [51]. Development of antibiofilm agents is very important as attempting to simply kill bacteria using antimicrobial treatment only is often insufficient when dealing with infections from biofilm-forming microbes such as *P. aeruginosa*. Antibiofilm agents may influence biofilm formation by damaging microbial membrane structures, inhibiting peptidoglycan synthesis, and/or modulating quorum sensing [55]. The mechanism of inhibition of biofilm formation of the isolated compound against *P. aeruginosa* still remains to be elucidated. Phytochemicals such as steroids have been shown to associate with bacterial proteins and inhibit microbial adhesion, enzymes cell envelop and transport proteins [56, 57]. It is possible that chondrillasterol inhibited biofilm formation through some of these mechanisms. One of the most significant features of bacterial biofilms is their resistance to antimicrobial agents [58]. Investigations into the mechanism of resistance revealed that, nutrient limitation, low bacterial growth rate and reduced drug penetration into the biofilms are all contributing factors [59].

Biofilm related infections can be efficiently eradicated by using antibiofilm agents that weaken or destroy the mature biofilm rendering cells to be susceptible to antibiotics [60]. The weakening or destruction of the mature biomass can be followed by washout or subsequent biocidal inactivation of the detached biomass [61, 62]. Chondrillasterol concentrations significantly disrupted mature biofilms of *P. aeruginosa*. The result suggests that the compound is an effective biofilm disruptor which can be used to control *P. aeruginosa* infections. A biofilm disrupting agent may increase penetration of antibiotics in the biofilm [63]. This is very important in reducing bacteria drug tolerance as it has been proposed that antibiotics that penetrate more slowly may give time for an adaptive phenotypic response that could potentially increase tolerance [63, 64]. Bacteria in biofilms have been shown to behave differently which make it difficult for the immune system to recognise and fight them [65, 66]. However, once a biofilm disruptor have been administered, cure can be obtained even without concurrent administration with antibiotics. This is because once the biofilm has been disrupted and cells are now in planktonic form then the cells will be exposed to vast immune intervention mechanisms produced by host against the pathogens [67]. Chondrillasterol may be used as an agent for disrupting biofilms and this may significantly diminish morbidity associated with biofilm related nosocomial infection [68]. Effective treatments for the disruption of established biofilms could save many lives and decrease healthcare costs related to the treatment and potential replacement of infected implanted prosthetic devices [69].

## Conclusion

Chondrillasterol was isolated from *V. adoensis* leaves. The compound has antibacterial activity against *P. aeruginosa S. aureus* and *K. pneumoniae*. Chondrillasterol inhibited biofilm formation in *P. aeruginosa* as well as disrupted already formed mature biofilms. Therefore, this compound may be useful in the development of antibacterial agents to treat biofilm-related infections.

### Recommendations

Further work is required to evaluate the mechanism of action of the compound as an antibiofilm agent as well as evaluation of its antibacterial potential and mechanism of action on a number of pathogens. The toxicity profile of the compound also needs to be evaluated. Further isolation and purification of fractions from other parts of this plant are recommended which could yield some novel bioactive compounds.

## Additional file

Additional file 1: **Figure S1.** NMR spectra of the various 2D experiments including H, H-COSY, HSQC, HMBC and NOESY used for structure elucidation of chondrillasterol. (PDF 1870 kb)

## Data Availability

The datasets used and/or analysed during the current study available from the corresponding author on reasonable request.

## Abbreviations

ATCC: American Type Culture Collection
MTT: 3-(4,5-dimethylthiazol-2-yl)-2, 5-diphenyltetrazolium bromide
TLC: Thin layer chromatography
