# Supplementary material for: Evaluation of antimicrobial activity of chondrillasterol isolated from Vernonia adoensis (Asteraceae)
Source: BMC Complement Altern Med. 2019 Sep 6;19:249. doi: 10.1186/s12906-019-2657-7 (PMC6731578; doi:10.1186/s12906-019-2657-7)
Supplement: Supplementary file 1 — Figure S1. NMR spectra of the various 2D experiments including H, H-COSY, HSQC, HMBC and NOESY used for structure elucidation of chondrillasterol. (PDF 1870 kb) [file 12906_2019_2657_MOESM1_ESM.pdf]

F10

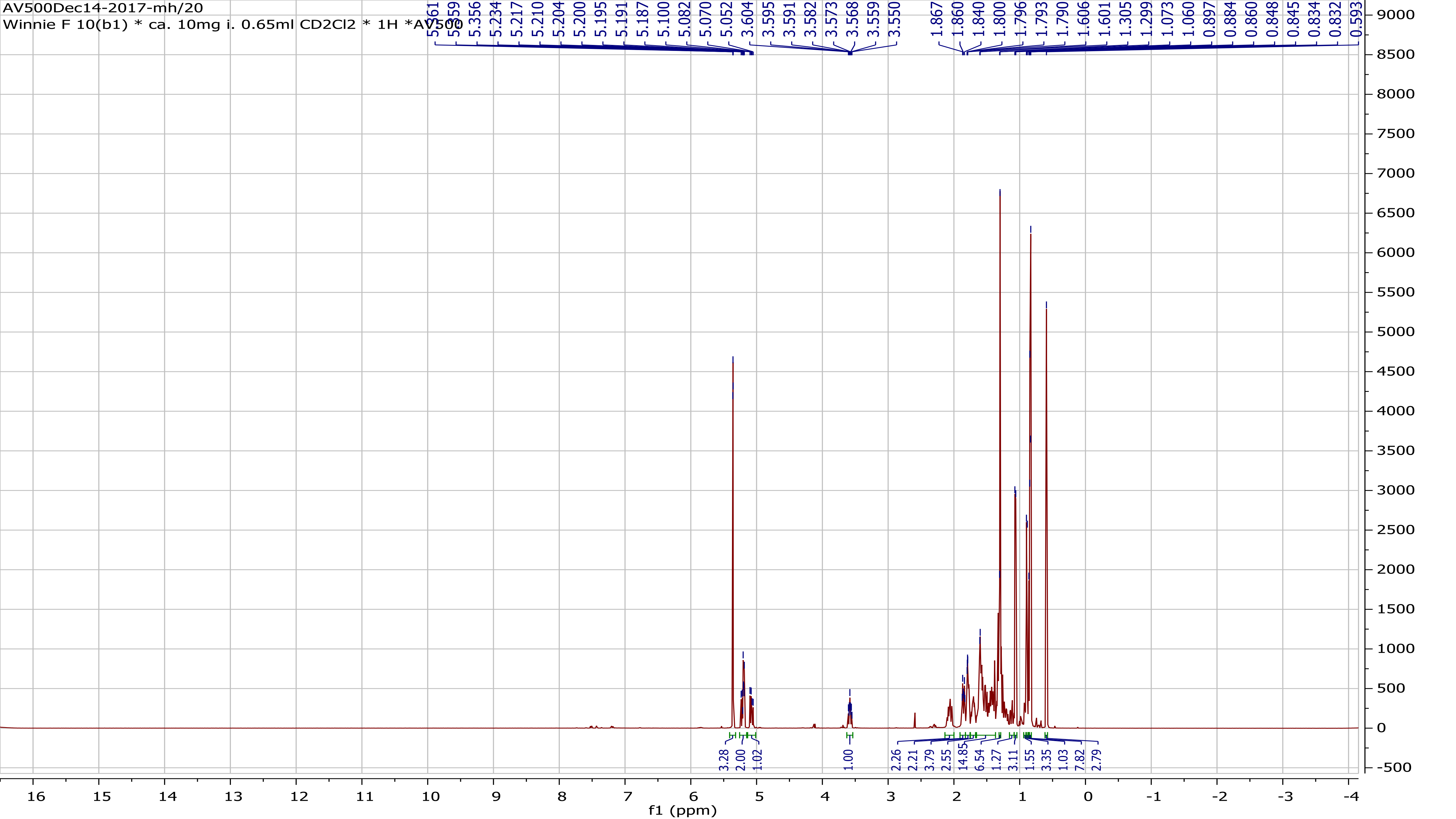
AV500Dec14-2017-mh/20
Winnie F 10(b1) * ca. 10mg i. 0.65ml CD2Cl2 * 1H *AV500
5.361 5.359 5.356 5.234 5.217 5.210 5.204 5.200 5.195 5.191 5.187 5.100 5.082 5.070 5.052 3.604 3.595 3.591 3.582 3.573 3.568 3.559 3.550 1.867 1.860 1.840 1.800 1.796 1.793 1.790 1.606 1.601 1.305 1.299 1.073 1.060 0.897 0.884 0.860 0.848 0.845 0.834 0.832 0.593
3.28 2.00 1.02 1.00 2.26 2.21 3.79 2.55 14.85 6.54 1.27 3.11 1.55 3.35 1.03 7.82 2.79
f1 (ppm)

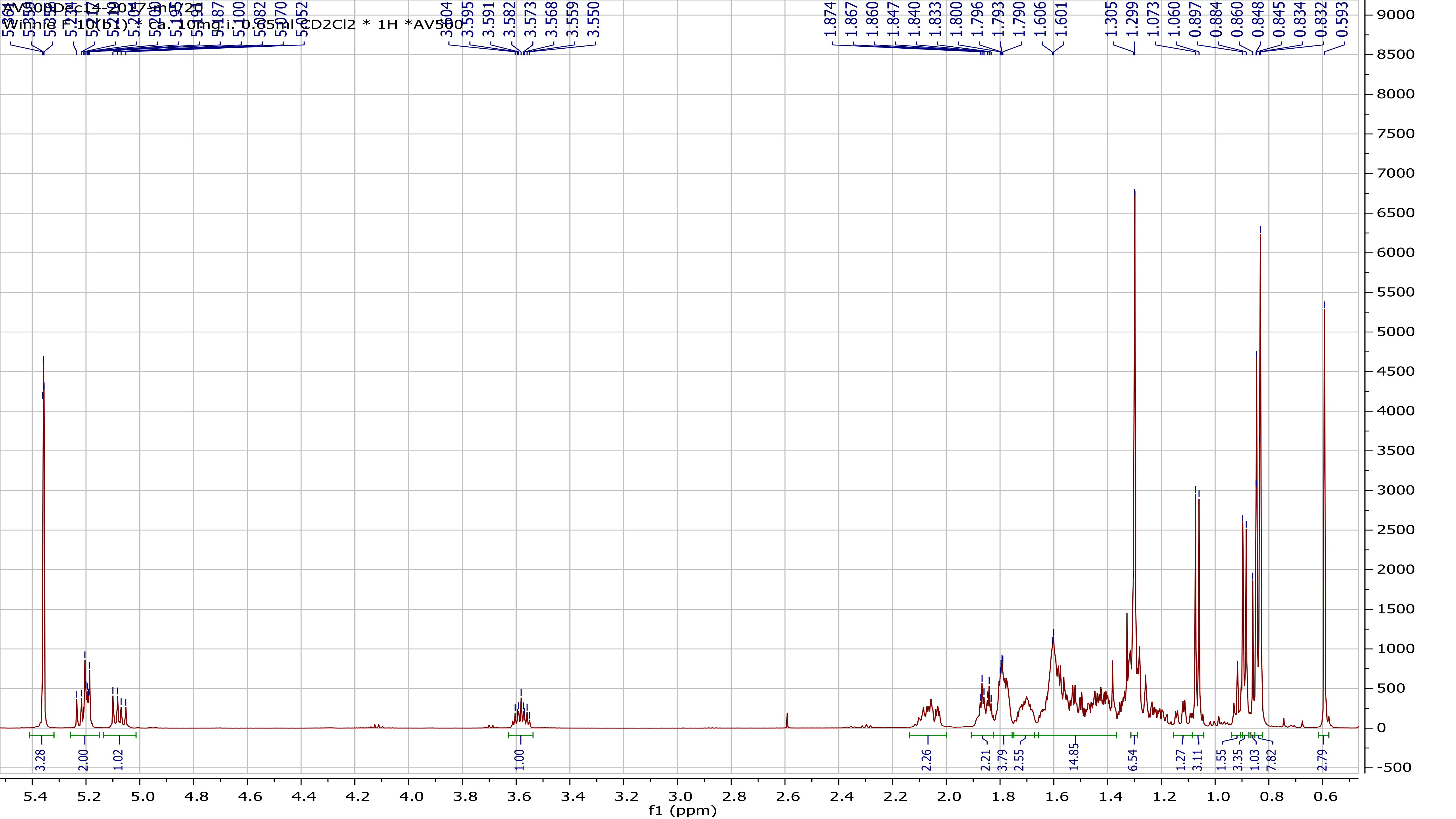
Winnie F.10(b1) * ca. 10mg i. 0.65ml CD2Cl2 * 1H *AV500
3.604
3.595
3.591
3.582
3.573
3.568
3.559
3.550
5.100
5.082
5.070
5.052
1.874
1.867
1.860
1.847
1.840
1.833
1.800
1.796
1.793
1.790
1.606
1.601
1.305
1.299
1.073
1.060
0.897
0.884
0.860
0.848
0.845
0.834
0.832
0.593
3.28
2.00
1.02
1.00
2.26
2.21
3.79
2.55
14.85
6.54
1.27
3.11
1.55
3.35
1.03
7.82
2.79
f1 (ppm)

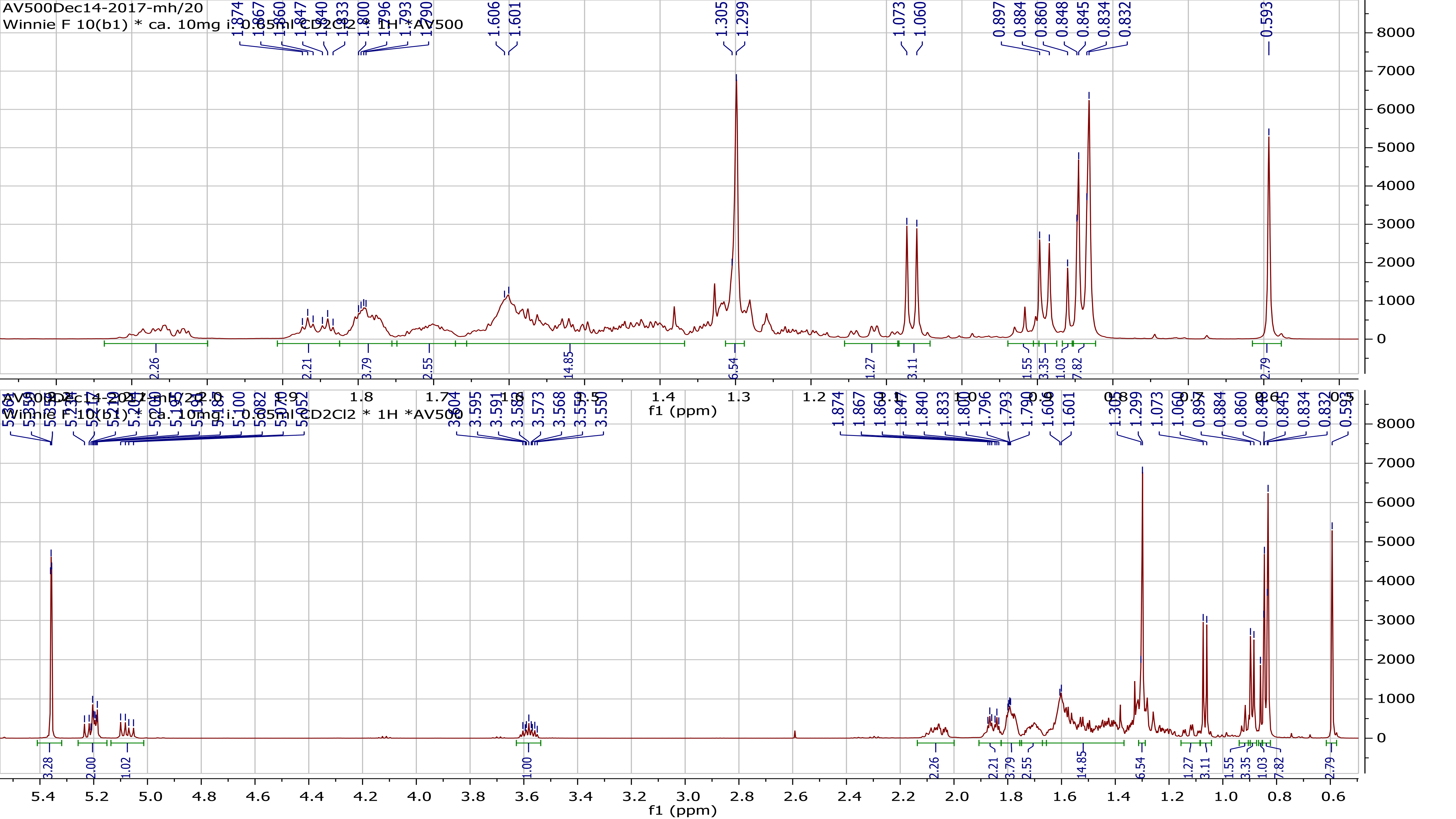
AV500Dec14-2017-mh/20
Winnie F 10(b1) * ca. 10mg i. 0.65ml CD2Cl2 * 1H *AV500
f1 (ppm)

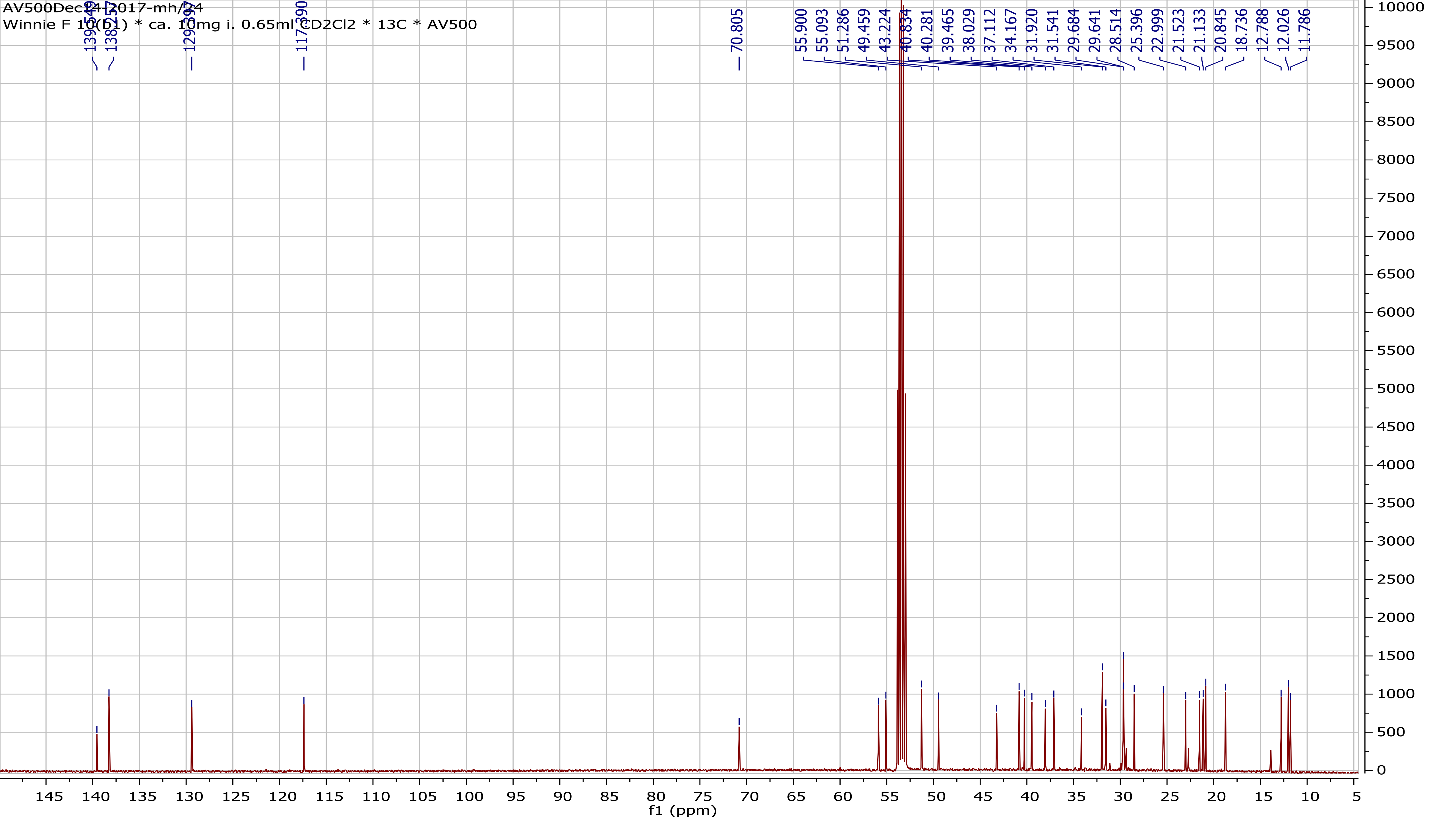

Winnie F 10(b1) * ca. 10mg i. 0.65ml CD2Cl2 * 13C * AV500
70.805
55.900
55.093
51.286
49.459
43.224
40.834
40.281
39.465
38.029
37.112
34.167
31.920
31.541
29.684
29.641
28.514
25.396
22.999
21.523
21.133
20.845
18.736
12.788
12.026
11.786
f1 (ppm)

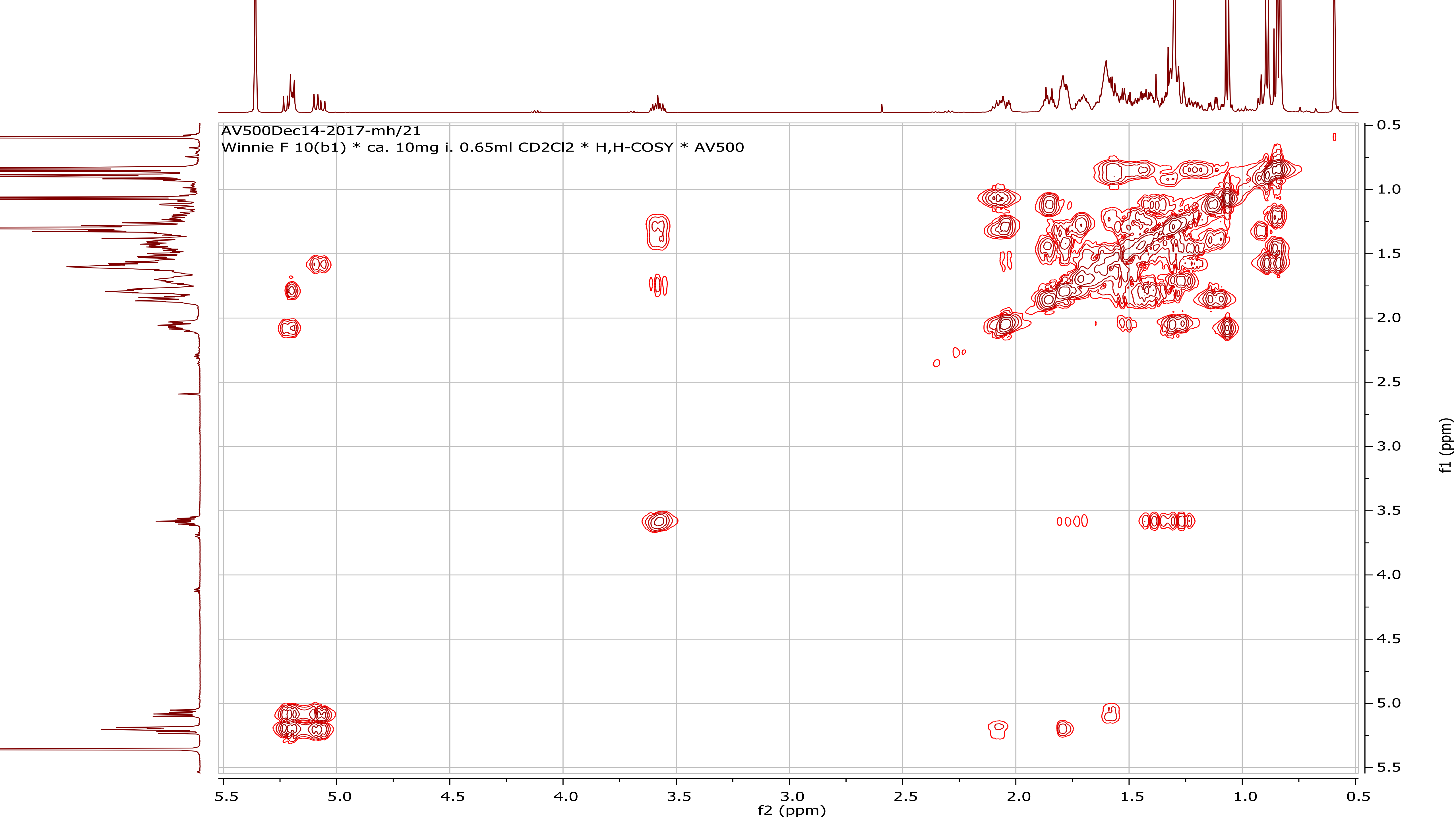
AV500Dec14-2017-mh/21
Winnie F 10(b1) * ca. 10mg i. 0.65ml CD2Cl2 * H,H-COSY * AV500
f2 (ppm)
f1 (ppm)
5.5
5.0
4.5
4.0
3.5
3.0
2.5
2.0
1.5
1.0
0.5

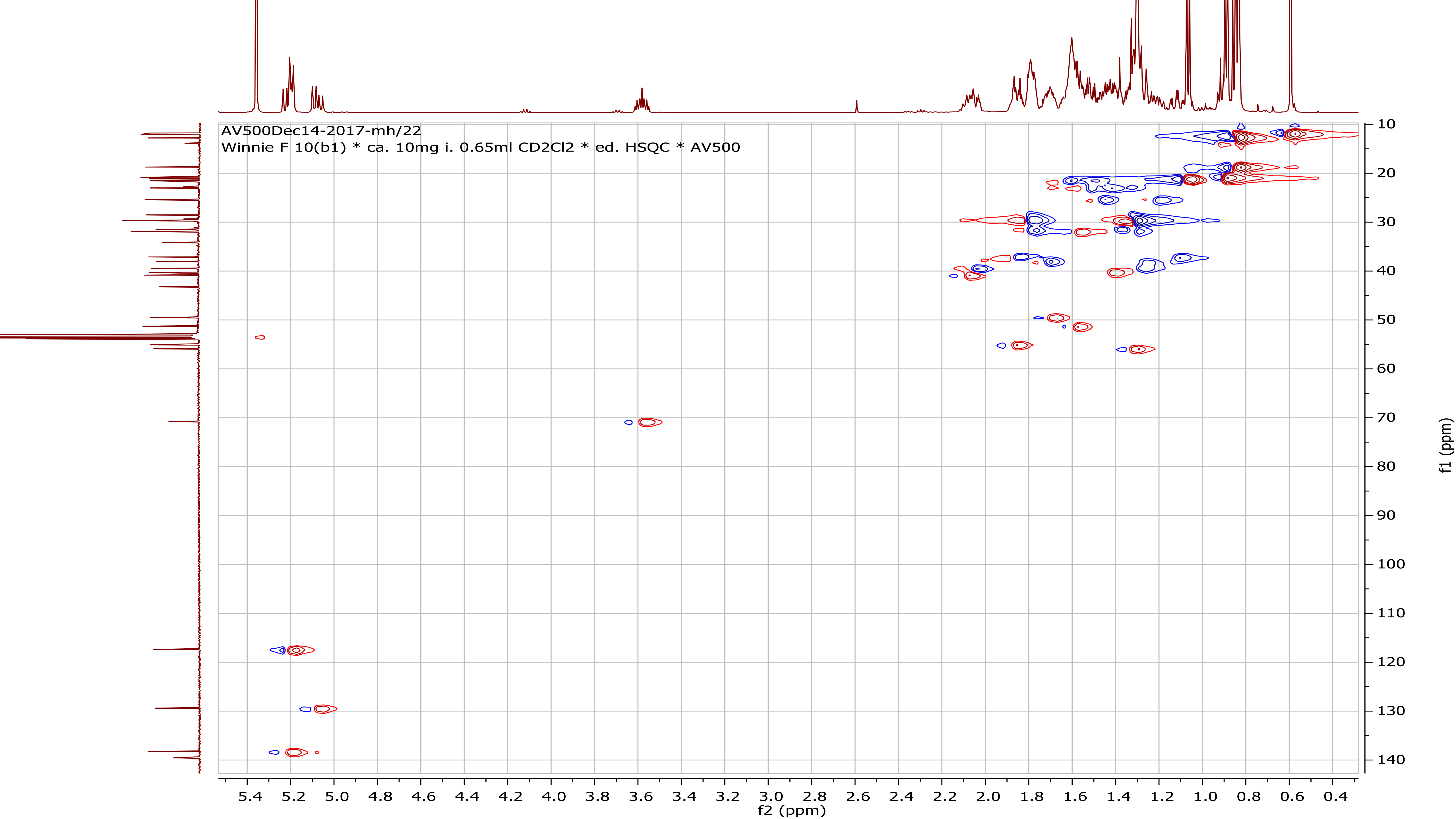
AV500Dec14-2017-mh/22
Winnie F 10(b1) * ca. 10mg i. 0.65ml CD2Cl2 * ed. HSQC * AV500
5.4 5.2 5.0 4.8 4.6 4.4 4.2 4.0 3.8 3.6 3.4 3.2 3.0 2.8 2.6 2.4 2.2 2.0 1.8 1.6 1.4 1.2 1.0 0.8 0.6 0.4
f2 (ppm)
10 20 30 40 50 60 70 80 90 100 110 120 130 140
f1 (ppm)

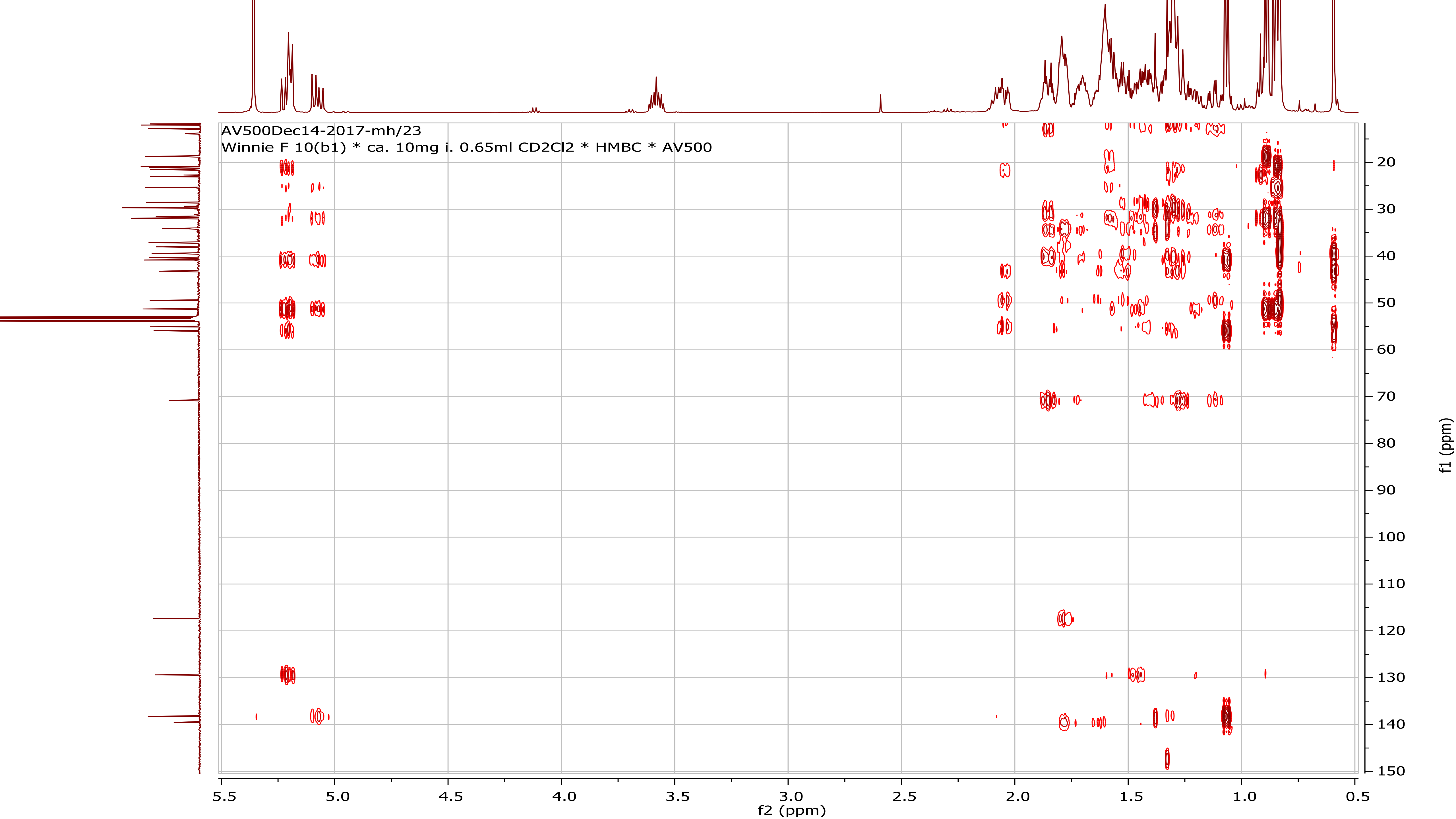
AV500Dec14-2017-mh/23
Winnie F 10(b1) * ca. 10mg i. 0.65ml CD2Cl2 * HMBC * AV500
f2 (ppm)
f1 (ppm)

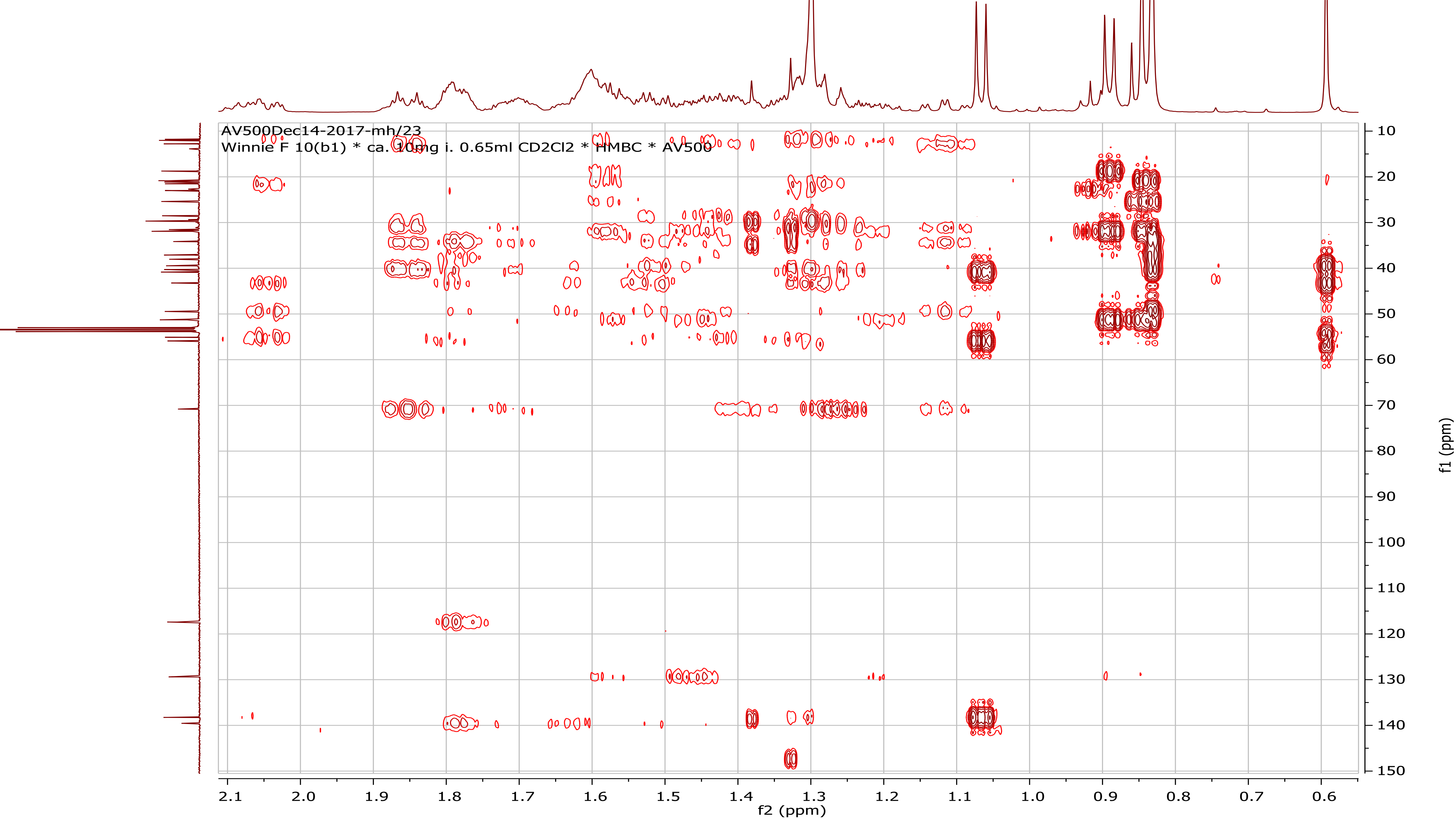
AV500Dec14-2017-mh/23
Winnie F 10(b1) * ca. 10mg i. 0.65ml CD2Cl2 * HMBC * AV500
f2 (ppm)
f1 (ppm)
2.1
2.0
1.9
1.8
1.7
1.6
1.5
1.4
1.3
1.2
1.1
1.0
0.9
0.8
0.7
0.6
10
20
30
40
50
60
70
80
90
100
110
120
130
140
150

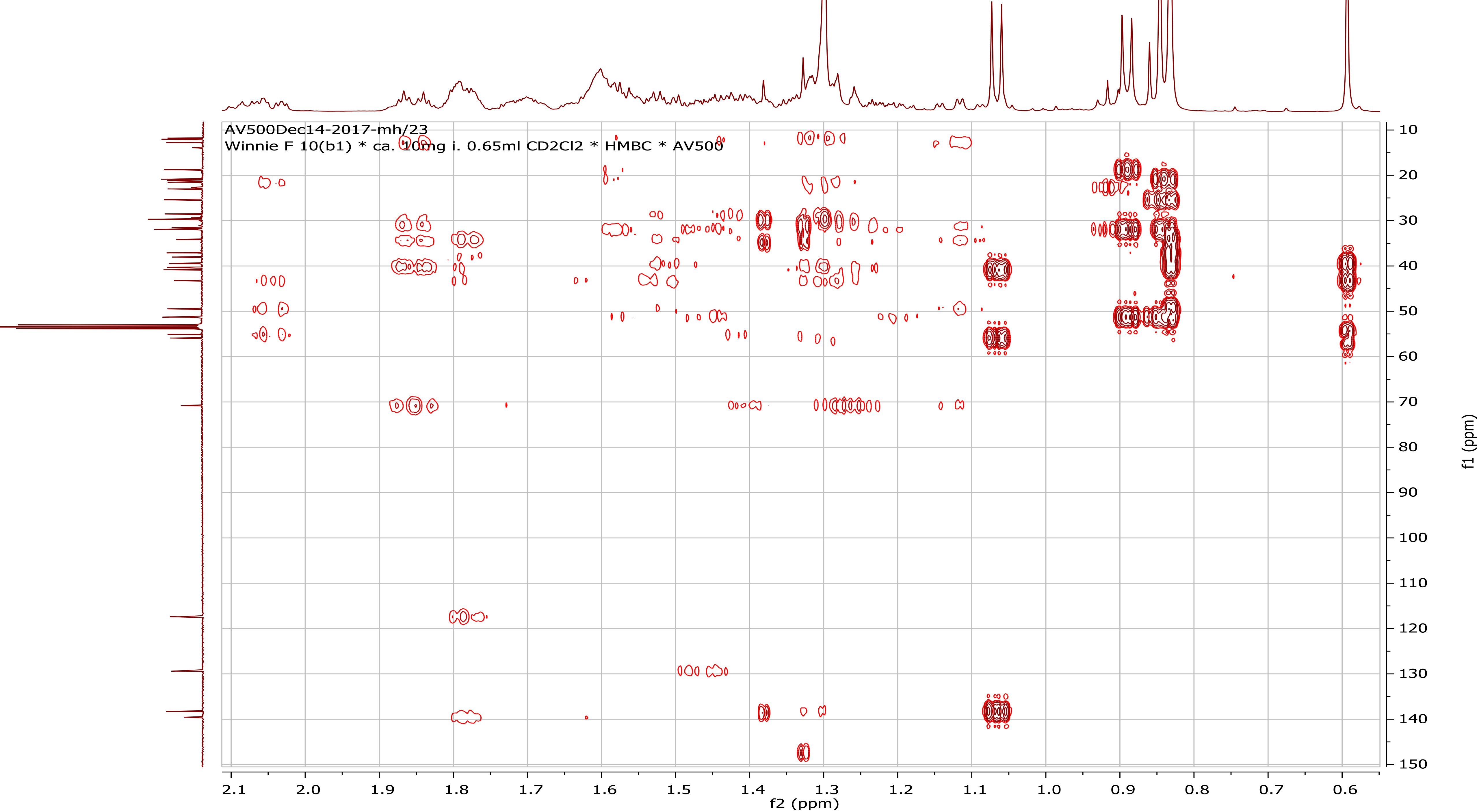
AV500Dec14-2017-mh/23
Winnie F 10(b1) * ca. 10mg i. 0.65ml CD2Cl2 * HMBC * AV500
f2 (ppm)
f1 (ppm)

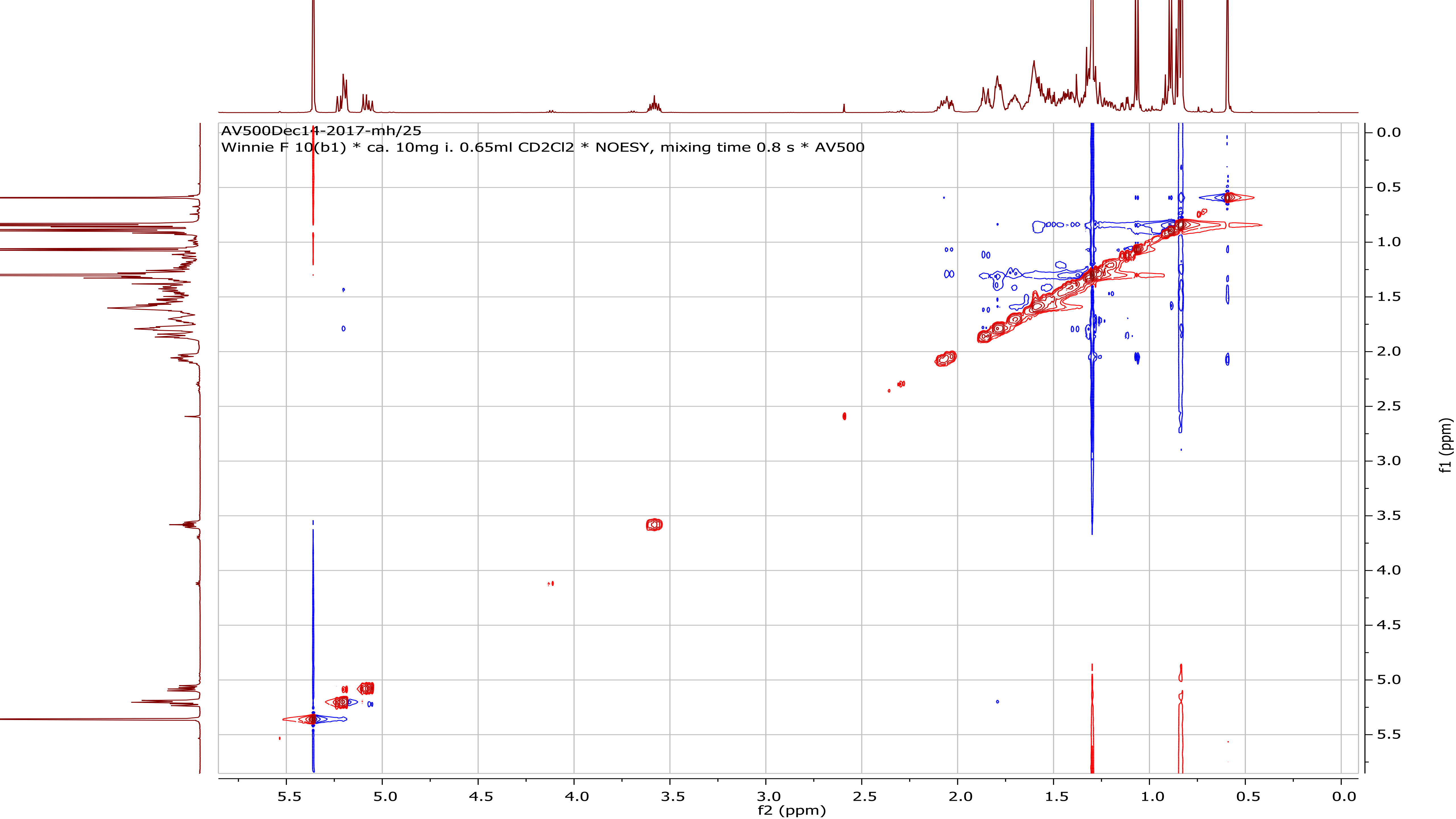
AV500Dec14-2017-mh/25
Winnie F 10(b1) * ca. 10mg i. 0.65ml CD2Cl2 * NOESY, mixing time 0.8 s * AV500
f2 (ppm)
f1 (ppm)

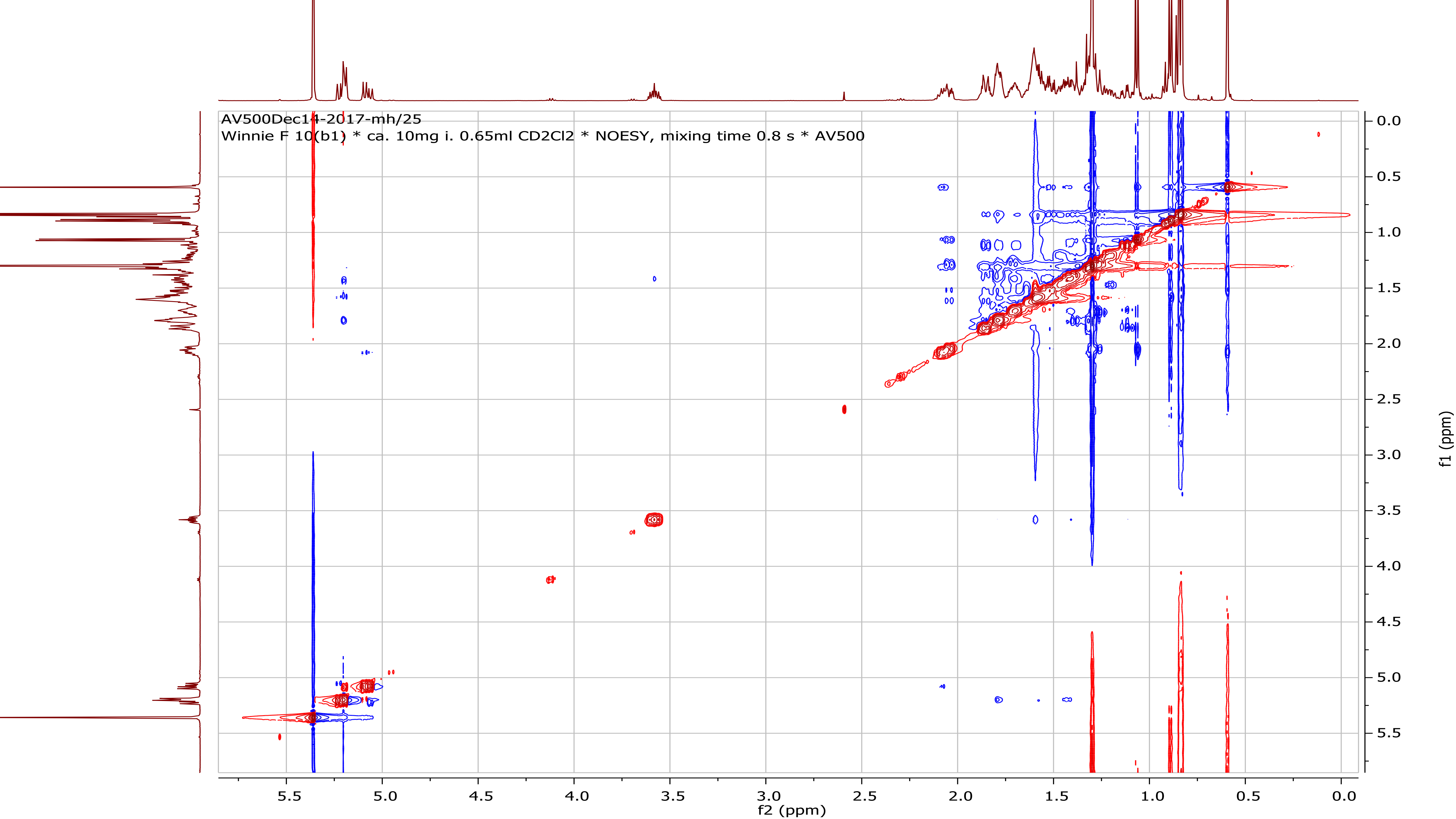
AV500Dec14-2017-mh/25
Winnie F 10(b1) * ca. 10mg i. 0.65ml CD2Cl2 * NOESY, mixing time 0.8 s * AV500
f2 (ppm)
f1 (ppm)
